# Predictors of response to bDMARDs and tsDMARDs in psoriatic arthritis: a pilot study on the role of musculoskeletal ultrasound

**DOI:** 10.3389/fmed.2024.1482894

**Published:** 2024-12-23

**Authors:** Giacomo Cozzi, Laura Scagnellato, Mariagrazia Lorenzin, Antonio Collesei, Francesca Oliviero, Amelia Damasco, Chiara Cosma, Daniela Basso, Andrea Doria, Roberta Ramonda

**Affiliations:** ^1^Rheumatology Unit, Department of Medicine-DIMED, University - Padova University Hospital, Padua, Italy; ^2^Cancer Genomics Core-Lab, Istituto Oncologico Veneto IRCCS, Padua, Italy; ^3^Laboratory Medicine, Department of Medicine-DIMED, University - Padova University Hospital, Padua, Italy

**Keywords:** psoriatic arthritis, bDMARDs, tsDMARDs, ultrasound, therapy response

## Abstract

**Objectives:**

This pilot study aimed to identify early predictors of drug retention in patients with clinically active peripheral psoriatic arthritis who initiated or switched to therapy with biologic and targeted synthetic disease-modifying antirheumatic drugs (bDMARDs and tsDMARDs).

**Methods:**

Clinical and ultrasound assessments were conducted at baseline (t0) and subsequently at 1 (t1), 3 (t3), and 6 (t6) months. Ultrasound evaluations targeted joints/entheses according to PsASon-Score13 and the most clinically involved joint/enthesis/tendon or the two most clinically involved joints/entheses/tendons (MIJET and 2MIJET). After 6 months of follow-up, patients were divided into two groups based on drug retention, determined by the clinician's assessment of treatment efficacy (cResponder vs. non-cResponder). Main endpoints were ultrasound changes in MIJET, 2MIJET, and GUIS (Global US Inflammation Subscore) derived from PsASon-13.

**Results:**

Twenty-nine patients were enrolled, 22 cResponders and 7 non-cResponders at t6. In the comparison between cResponders and non-cResponders, GUIS variation significantly differed in Δt6-t0, while MIJET and 2MIJET variations were significant as early as Δt3-t0 and confirmed in Δt6-t0. The ultrasound response of MIJET and 2MIJET was faster in cResponders treated with JAKi vs. those treated with TNFi and IL-17/12-23i, significant in Δt1-t0.

**Conclusions:**

Ultrasound imaging of clinically involved joint sites may be a valuable early predictor of therapy response for predicting drug retention at 6 months in patients with psoriatic arthritis.

## 1 Introduction

Psoriatic arthritis (PsA) is a complex condition that still presents several unmet needs. Researchers have highlighted key areas of investigation in PsA, including tests to improve the assessment of disease activity and treatment response (1). The development of early predictors of treatment response would help tailor treatment to each patient and avoid inappropriate therapies or unnecessary therapeutic switches (2, 3).

The treatment of PsA includes conventional synthetic disease-modifying antirheumatic drugs (csDMARDs), biological therapies (bDMARDs), and orally administered targeted small molecule drugs (tsDMARDs). Despite the availability of numerous drugs, ~40% of patients fail to respond to csDMARDs or bDMARDs (4).

Serum biomarkers have been studied as possible predictors of treatment response. Elevated levels of C-reactive Protein (CRP) are found early in 33–89% of PsA patients; thus, there is a significant percentage of patients without inflammation despite active disease (1). This is likely due to the fact that in SpA, joint and entheseal damage driven by local cytokines does not fully reflect the systemic context (5).

Among imaging techniques, ultrasound (US) is the most promising for the assessment of treatment response, as it is safer, easily accessible and time efficient. Despite numerous studies published on the use of US in PsA, its application in clinical practice remains a matter of debate (6, 7). A recent systematic literature review comprising 15 studies evaluated the role of US in the follow-up and prognosis of PsA. A major limitation that emerged was the variability in the definitions of elementary lesions and US scoring systems. Five different scoring systems were used across the selected articles (8), two of which considered different domains involved in PsA (joints, entheses, and tendons): (i) the Five Targets PwD score for psoriasis; (ii) the composite US score for evaluating inflammatory and structural pathology in PsA, including 13 and 22 joints (PsASon-Score 13 and 22, respectively) (9, 10). Both composite scores showed sufficient sensitivity to detect US variations following therapy. Moreover, it has recently been proposed to also use in patients with PsA the global synovitis scoring system EULAR-OMERACT “GLOESS,” which was developed in patients with rheumatoid arthritis (RA) (11). Although this scoring system allows to globally assess synovial severity across numerous joints, it does not evaluate the involvement of entheses and tendons. Thus, there are two main unmet needs when discussing the role of US in PsA follow-up. The first is to find a US score allowing us to monitor disease activity in all its domains, both in clinical practice and in clinical studies. The second is to find US predictors of treatment response. There are indeed few studies on the US response in PsA treatment.

In this context, the primary endpoint of our pilot study was to identify early US predictors of drug retention with bDMARDs/tsDMARDs at 6 months. Thus, we recruited patients with active PsA who initiated therapy with bDMARDs/tsDMARDs, or who switched/swapped to other bDMARDs/tsDMARDs (due to ongoing treatment failure). The secondary endpoints of this study were: (i) to identify differences between clinical and ultrasound responses; (ii) to identify differences in sonographic scores among patients treated with different therapeutic classes.

## 2 Methods

### 2.1 Patients and study design

In this prospective observational study, patients with peripheral involvement of PsA, either treatment-naïve or with inadequate response to bDMARDs, were recruited. At baseline (*t0*), they presented clinically active arthritis and/or enthesitis, warranting initiation of bDMARDs/tsDMARDs therapy. The patients were followed at a rheumatology outpatient clinic located in northern Italy specializing in SpA treated with biological therapy.

Inclusion criteria were: (i) Age > 18 years; (ii) Subjects affected by PsA [according to CASPAR criteria (12)] with peripheral involvement; (iii) at least one of: (a) patients not treated with bDMARDs who, according to the clinician, must initiate bDMARDs; (b) patients who switch to/swap to another bDMARD as indicated by the treating rheumatologist; (iv) stable treatment for at least 12 weeks before treatment modification; (v) at least one of the following: (a) swollen joint count (SJC) ≥1 and tender joint count (TJC) ≥1; (b) ≥1 dactylitis; (c) Leeds enthesitis index (LEI) score ≥1; (d) clinical tendonitis/tenosynovitis in ≥1 sites.

Exclusion criteria were: (i) minimal disease activity (MDA) (13) at baseline; (ii) patients who are unable or unwilling to attend follow-up visits or provide accurate or consistent information about their symptoms and medical history.

Approval for the study was obtained from our institution's ethics committee (n. 52,723), and all participants provided informed consent according to the principles of the Declaration of Helsinki.

### 2.2 Clinical, US, and biochemical assessment

Clinical and US assessments were performed at baseline (t0) and subsequently at 1 (t1), 3 (t3), and 6 (t6) months from the treatment initiation.

US assessment was performed by a Rheumatologist with expertise in advanced musculoskeletal US (certification issued by the Italian Society of US in Medicine and Biology—SIUMB), using a Mylab X5 machine (Esaote Biomedica, Genoa, Italy) equipped with a linear transducer with 15–22 MHz frequency to investigate superficial articular-tendon regions; and a linear transducer with 4–15 MHz frequency to examine large joints and deep articular-tendon regions. Joints, entheses and tendons included in the unilateral PsASon13-Score (2 MCPs, 3 hand PIPs (H-PIP), 1 PIP of feet (F-PIP), 2 MTPs, 1 H-DIP, 2 F-DIPs, knee, wrist and the entheses of lateral epicondyle and distal patellar tendon) were evaluated.

Furthermore, if not already included in the aforementioned score, we also performed US of the most clinically involved joint/enthesis/tendon or the two most clinically involved joints/entheses/tendons (MIJET and 2MIJET, respectively) at baseline, according to the clinician. The grading and definition of synovitis and tenosynovitis were assessed according to the OMERACT score. Peritenonitis was graded in grayscale (GS) and powerDoppler (pD; 0 = absent; 1 = present). Erosions and osteophytes were semiquantitatively assessed (from 0 to 3). Enthesitis was defined and graded according to the Madrid sonographic enthesitis index (MASEI) with the addition of the evaluation of the lateral epicondyle (MASEI+E) in GS and pD, also assessing the presence of erosions and enthesophytes (14).

The main outcomes were the changes in the sum scores of synovitis/enthesitis/tenosynovitis of the MIJET, 2MIJET, and the GUIS (Global US Inflammation Subscore) derived from PsASon-13, considering only variations in GS and pD (10).

The clinical assessment of patients was conducted by two rheumatologists with expertise in SpA. We conducted a double-blind assessment of patients, as regards the clinicians and the ultrasonographer. The mean change from baseline was assessed for: TJ68, SJ66, psoriasis area severity Index (PASI), MDA, LEI, the clinical disease activity index for psoriatic arthritis (cDAPSA), psoriatic arthritis impact of disease (PSAID), health assessment questionnaire (HAQ). For each patient, anthropometric measurements and comprehensive medical history were recorded.

The biochemical assessment for patients included routine blood tests such as white blood cells count, liver and renal function tests, CRP and ESR.

### 2.3 Statistical analysis

Data were represented as mean (standard deviation) or median (interquartile range –IQR) as appropriate, for continuous variables. Categorical variables were expressed as number (percentage). Between-group comparisons of normally distributed variable values were performed using Student's *t*-test, to compare both the baseline characteristics of the patients and the means of the variations between t1/t3/t6 and t0. The chi-square test was used to explore the significance of observed differences for categorical variables. The differences between the trends observed in the mean values of clinimetric and ultrasound indices were assessed using the Cochran-Armitage test. All statistical analyses were carried out with the GraphPad prism 9.0 software (GraphPad Software, San Diego, CA, USA).

## 3 Results

### 3.1 Baseline demographic and clinical characteristics

Twenty-nine consecutive patients were enrolled (14 male, 48.3%), with a mean age of 57.72 (±9.72) years. The demographic, clinical, serological and ongoing therapy data prior to the switch/swap at *t0* are summarized in Supplementary Table 1. Seven (24%) patients were smokers vs. 5 (17.2%) who had quit smoking for more than 5 years. Most patients had failed one or more lines of bDMARDs, whereas 6 (20.7%) were naïve to bDMARDs. Patients had a high baseline disease activity with a mean cDAPSA of 28.16 (±11.88) and an average disease duration of 154 (±101.85) months. Twelve patients (41%) presented onychopathy.

The distribution of MIJET included small joints of the hands (MCP, PIP, DIP; *n* = 13), large joints (wrist, knee, ankle; *n* = 10), and entheses/tendons (epicondyle, Achilles enthesis and tendon, peroneal tendon; *n* = 6). For patients who had more than one joint involved at *t0*, for 2MIJET assessment, small joints of the hands (MCP, PIP, DIP; *n* = 13), large joints (elbow, wrist, shoulder; *n* = 4), and entheses/tendons (epicondyles, Achilles enthesis; *n* = 5) were considered.

“After 6 months of follow-up, clinicians assessed treatment efficacy and patients were divided into two groups based on drug retention: 22 patients continued therapy (cResponders), whereas 7 discontinued treatment at t6 due to clinician-assessed therapy failure (non-cResponders).”

The baseline characteristics of cResponders were compared to non-Responders. No significant differences were found between the two groups as it relates to age, sex, disease duration, BMI, onychopathy, TJC, SJC, PASI, cDAPSA, HAQ, PsAID, and naïve/non-naïve for bDMARD (*p* > 0.05 for all comparisons; Supplementary Table 2). Conversely, there was a significant difference in the mean LEI between the two groups (0.41 vs. 2.29, *p* = 0.0095).

### 3.2 Correlation between therapy response and US and clinical indices

The mean changes in clinimetric indices were compared over time intervals *t1*-*t0* (Δ*t1-t0*), *t3*-*t0* (Δ*t3-t0*), and *t6*-*t0* (Δ*t6-t0*) between cResponders and non-cResponders (Table 1). The difference in mean cDAPSA variations was not significant in the Δ*t1-t0* and Δ*t3-t0* intervals, whereas it was significant in Δ*t6-t0*. The comparison between the mean variations of HAQ and PsAID clinical indices was not significant. The Δ*t6-t0* difference in mean GUIS variations showed a significant difference in cResponders vs. non-cResponders, unlike Δ*t1-t0* and Δ*t3-t0*. The comparison of the variation of the MIJET US score in cResponders compared to non-cResponders showed a statistically significant difference in Δ*t3-t0* and Δ*t6-t0* (Δ*t3-t0*:−1.727 vs. 0,0; *p* = 0.00236; Δ*t6-t0*:−1.864 vs. 0.1429, *p* = 0.0055) but was not significant in Δ*t1-t0*. Similarly, for the 2MIJET, a statistically significant difference was evident in Δ*t3-t0* and Δ*t6-t0* and was not significant in Δ*t1-t0*. In the sub-analysis of the 2MIJET evaluation, variations in GS (2MIJET-GS) and pD signal (2MIJET-pD) were separately assessed. A statistically significant difference was found for the 2MIJET-GS variations in Δ*t3-t0* and Δ*t6-t0* between cResponders and non-cResponders (Δ*t3-t0*:−1.733 vs.−0.2857, *p* = 0.0063; Δ*t6-t0*:−1.773 vs. 0.5714; *p* = 0.0008) and was not significant in Δ*t1-t0*. However, variations in 2MIJET-pD were not significant in the same time intervals.

**Table 1:** The mean variations of values between t1-t0, t3-t0, and t6-t0 among cResponder patients compared to non-cResponder patients.

	Δ**t1-t0**			Δ**t3-t0**			Δ**t6-t0**		
	**cResponder**	**Non-cResponder**	***p*** **-value**	**cResponder**	**Non-cResponder**	***p*** **-value**	**cResponder**	**Non-cResponder**	***p*** **-value**
**GUIS**	−1.909	0.7143	0.0634	−3.682	0.4286	0.0528	−5.136	1.000	**0.0231** ^*^
**MIJET**	−0.9545	−0.1429	0.2482	−1.727	0.000	**0.0236** ^*^	−1.864	0.1429	**0.0055** ^*^
**2MIJET**	−1.682	−0.5714	0.2582	−3.227	−0.7143	**0.0497** ^*^	−3.409	0.1429	**0.0030** ^*^
**2MIJET GS**	−0.8182	0.000	0.1480	−1.773	0.2857	**0.0063** ^*^	−1.773	0.5714	**0.0008** ^*^
2MIJET pD	−0.8636	−0.5714	0.6324	−1.636	−0.5714	0.1467	−1.636	−0.2857	0.0612
**cDAPSA**	−10.27	−8.571	0.6496	−11.59	−9.286	0.5581	−12.86	−4.286	**0.0256** ^*^
HAQ	−0.1655	−0.1786	0.9219	−0.2950	−0.3036	0.9626	−0.2491	−0.1786	0.6448
PsAID	−0.9080	−1.179	0.2686	−1.476	−1.329	0.8319	−1.619	−1.071	0.3441

GUIS, Global US Inflammation Subscore; MIJET, Most Involved Joint/Enthesis/Tendon; 2MIJET, two Most Involved Joints/Entheses/Tendons; GS, Gray Scale; pD, powerDoppler; cDAPSA, clinical Disease Activity in PSoriatic Arthritis; HAQ, Health Assessment questionnaire; PsAID, Psoriatic Arthritis Impact of Disease; cResponder, clinical Responder.

^*^Statistically significant variations.

**Figure 1:**
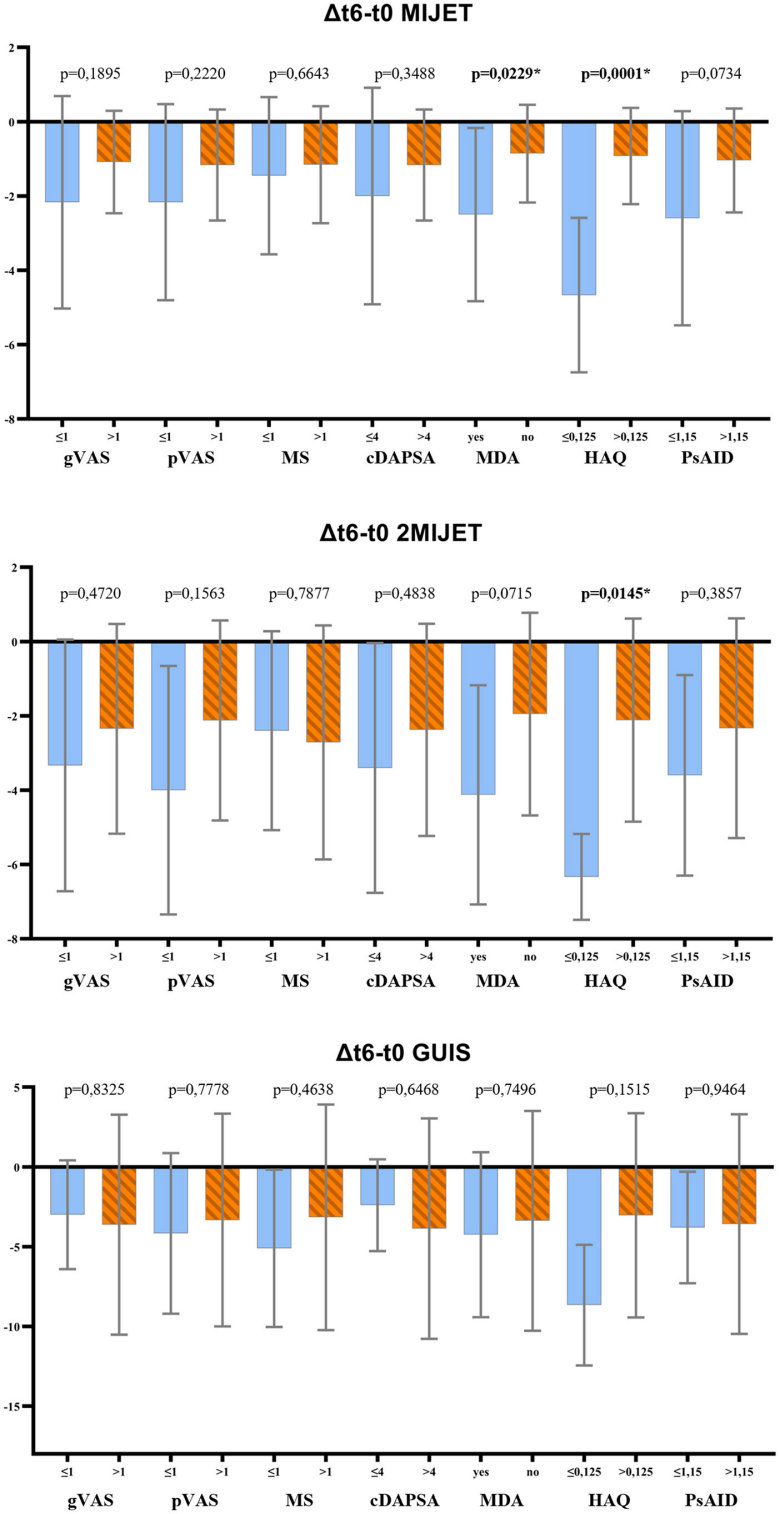
*t*-test results for the correlation between clinimetric indices and the variation in ultrasound scores at t6. MIJET, Most Involved Joint/Enthesis/Tendon; 2MIJET, two Most Involved Joints/Entheses/Tendons; GUIS, Global US Inflammation Subscore; gVAS, global assessment visual analog scale; pVAS, pain visual analog scale; MS, morning stiffness; cDAPSA, clinical disease activity index for psoriatic arthritis; MDA, minimal disease activity; HAQ, health assessment questionnaire; PsAID, psoriatic arthritis impact of disease. *Statistically significant variations.

At the end of the 6-month follow-up, the correlation between clinimetric and ultrasound indices was assessed to determine whether a disease deemed in clinical remission -i.e., achievement of MDA, cDAPSA ≤ 4—showed a statistically significant difference in the variation of ultrasound scores compared to patients who did not achieve remission. Similarly, the correlation between ultrasound indices, patient's quality of life (HAQ, PsAID) and patient-reported outcomes (PROs) of pain and morning stiffness at t6 was evaluated. The analysis revealed that among the PROs, only a good quality of life according to the HAQ (≤ 0.125) correlated significantly with the variation of the MIJET and 2MIJET ultrasound scores, but not with the GUIS. Among the composite disease activity indices cDAPSA and MDA, only the latter showed a significant correlation with the changes in the MIJET ultrasound score, but not with the 2MIJET and GUIS (Figure 1, Supplementary Table 3).

**Figure 2:**
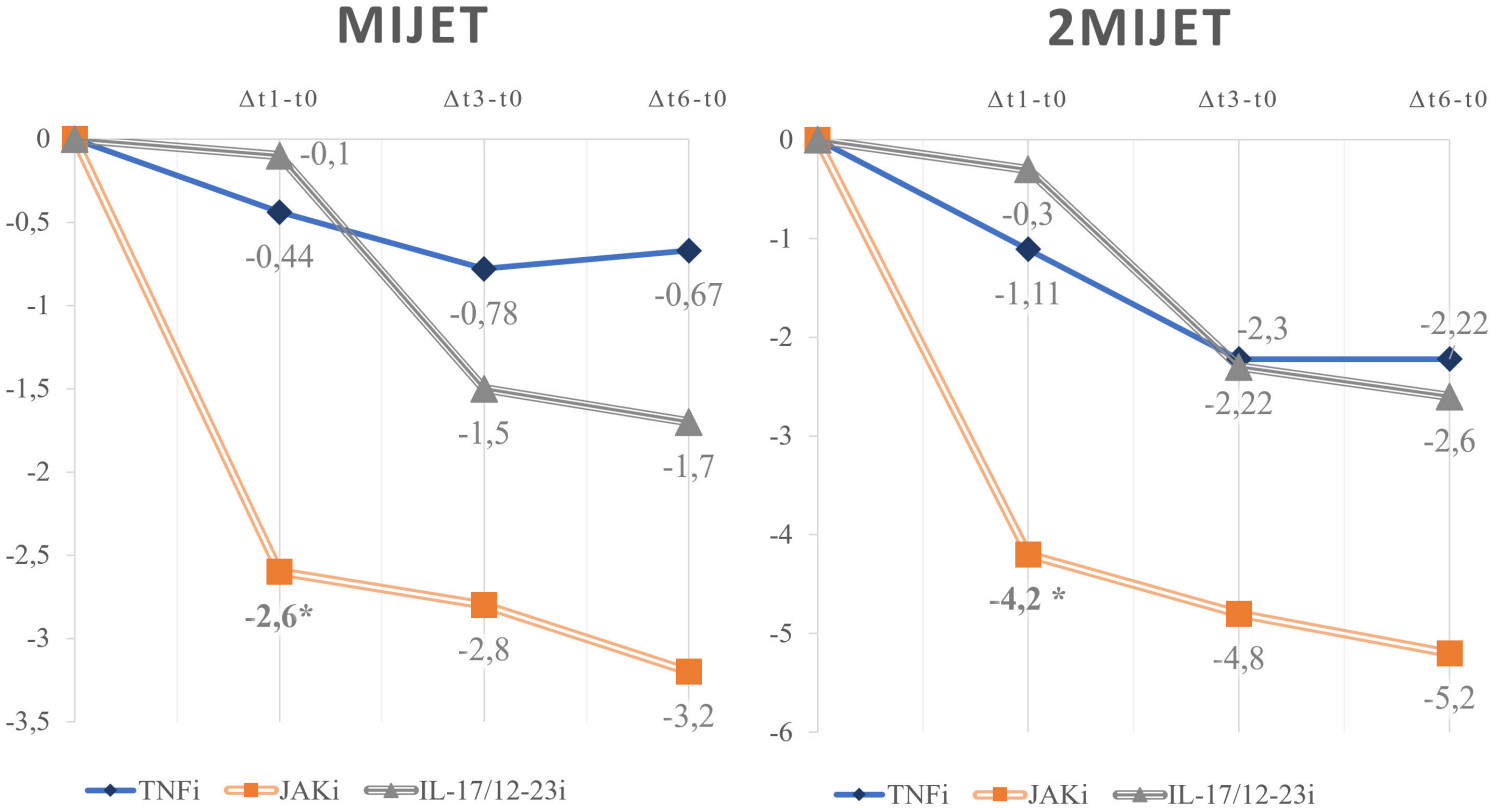
Density plots of the trends in the mean values of clinimetric and ultrasound indices between cResponders and non-cResponders at t0, t1, and t3. MIJET, Most Involved Joint/Enthesis/Tendon; 2MIJET, two Most Involved Joints/Entheses/Tendons; GUIS, Global US Inflammation Subscore; GUIS.e, Global US Inflammation Subscore enthesis; pVAS, pain visual analog scale; MS, morning stiffness; cDAPSA, clinical disease activity index for psoriatic arthritis; HAQ, health assessment questionnaire; PsAID, psoriatic arthritis impact of disease. *Statistically significant variations.

Furthermore, an analysis of trends observed in the mean values of clinimetric and ultrasound indices at t0, t1, and t3 revealed no significant differences between cResponders and non-cResponders as shown by the density plots in Figure 2 (Supplementary Table 4).

### 3.3 Variation of US and clinical indices in comparison to different therapies

**Figure 3:**
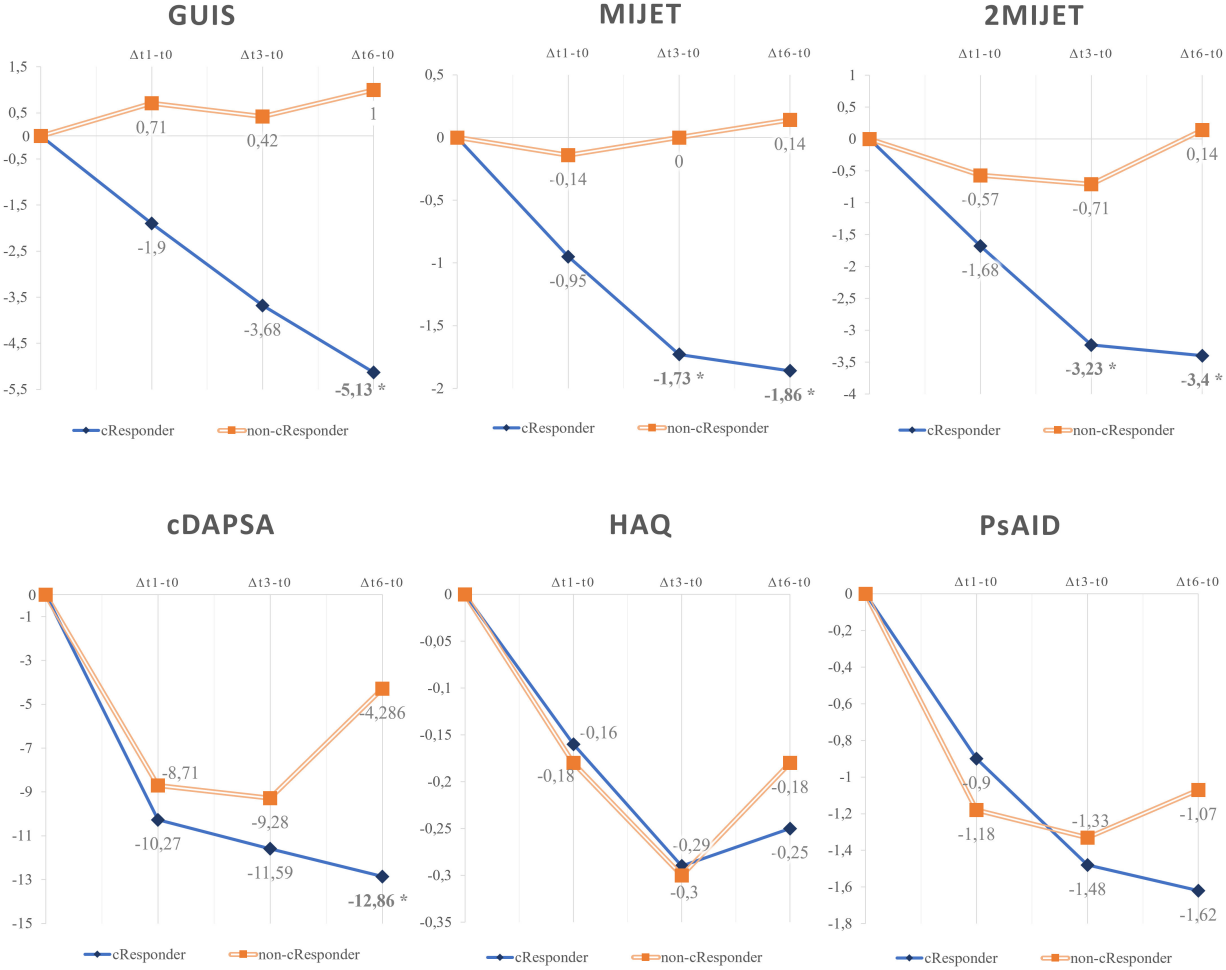
Graphical representation of the mean variations of MIJET and 2MIJET between t1-t0, t3-t0, and t6-t0 among c-Responder patients across different pharmacological classes. MIJET, Most Involved Joint/Enthesis/Tendon; 2MIJET, two Most Involved Joints/Entheses/Tendons. *Statistically significant variations.

Among c-Responder patients, the variations of US scores were compared in the intervals Δ*t1-t0*, Δ*t3-t0*, and Δ*t6-t0* across different pharmacological classes (Figure 3). Eight patients were treated with TNFi, 8 patients with interleukin inhibitors (IL-17i, *n* = 5; IL-12-23i, *n* = 3), and six patients with JAKi. Early variations in the mean difference of MIJET and 2MIJET in Δ*t1-t0* were statistically significant in the comparison between TNFi and JAKi (MIJET:−0.444 vs.−2.6, *p* = 0.0491; 2MIJET:−1.111 vs.−4.2, *p* = 0.0333). The same trend was confirmed for the comparison between IL-17/12-23i and JAKi in Δ*t1-t0* (MIJET:−0.1 vs.−2.6, *p* = 0.0194; 2MIJET:−0.3 vs.−4.2, *p* = 0.0021). However, these significant differences were not sustained in the intervals Δ*t3-t0* and Δ*t6-t0* (Supplementary Table 5).

### 3.4 Adverse events

During the observation period, some adverse events (AEs) were recorded, although they did not lead to treatment interruption but only temporary suspension of the therapy. No serious (S)AEs were recorded. Six infectious AEs (1 upper respiratory tract infection, 4 SARS-CoV2 viral infections, and 1 diverticulitis), and 2 hypertensive crises were reported.

## 4 Discussion

**Figure 4:**
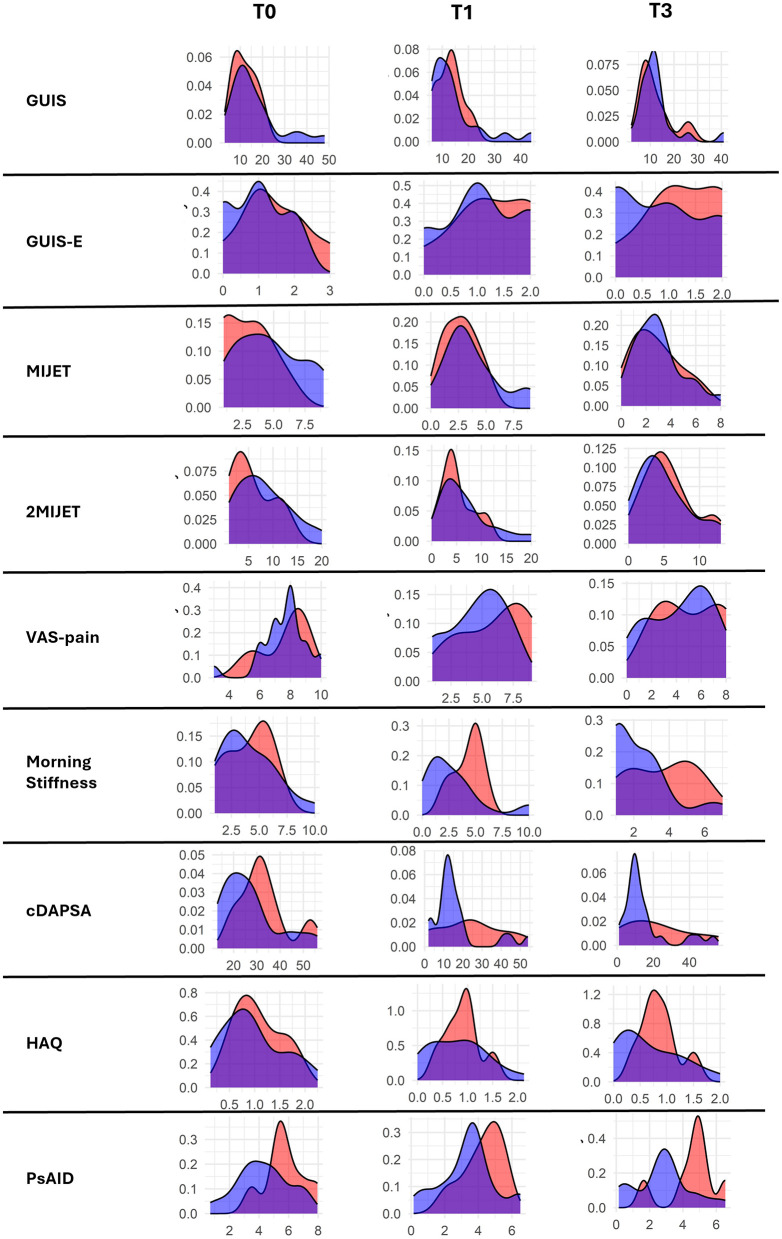
Graphical representation of the mean variations of values between t1-t0, t3-t0, and t6-t0 among cResponder patients compared to non-cResponder patients. GUIS, Global US Inflammation Subscore; MIJET, Most Involved Joint/Enthesis/Tendon; 2MIJET, two Most Involved Joints/Entheses/Tendons; HAQ, Health Assessment questionnaire; PsAID, Psoriatic Arthritis Impact of Disease; cDAPSA, clinical Disease Activity in PSoriatic Arthritis. *Statistically significant variations. *Statistically significant variations.

In this pilot study, we aimed to assess how US evaluations may help predict drug retention of bDMARDs and tsDMARDs therapies. For this purpose, the MIJET and 2MIJET ultrasound scoring systems have been proposed. By focusing on specific sites with a high prevalence of inflammatory activity, they enhance the integration of ultrasound with clinical examination in a time-efficient manner, potentially increasing specificity in detecting inflammatory activity within articular and peri-articular structures. Patients who continued therapy after 6 months, as deemed effective by clinicians, showed statistically significant improvement in the ultrasound assessment of the most clinically involved joints, MIJET and 2MIJET, as early as t3, with further confirmation at t6. In contrast, the variation in the unilateral GUIS ultrasound score was significant only at t6, identifying cResponders less quickly than the MIJET and 2MIJET score (Figure 4). Biologic therapy appeared to be associated with a faster ultrasound response compared to the clinical response. Indeed, the variation in clinical disease activity according to cDAPSA was not significant at t1 and t3 between cResponders and non-cResponders, unlike at t6. The HAQ and PsAID questionnaires did not show significant variations between cResponders and non-cResponders during the 6-month follow-up period, although a trend toward improvement in scores was observed among cResponders (Figure 4). This may be partly attributed to the relatively long average disease duration in our cohort, as well as the associated chronic damage and secondary pain that compromise the quality of life in these patients (15).

Some studies in the literature have previously confirmed the validity of ultrasound in assessing response to single biologic drug (16). A recent randomized controlled trial on 166 PsA patients demonstrated how changes in the US—according to the GLOESS US score—may appear as early as 1 week after initiating secukinumab therapy (17). It bears noting that the Authors also reported that the variation in GS of the GLOESS score was more sensitive vs. changes in the pD GLOESS, a finding that was corroborated in our study. Specifically, the variation in 2MIJET-GS, but not in 2MIJET-pD, was greater among cResponders vs. non-cResponders (Table 1). This could be partially explained by the lower baseline levels of the power Doppler signal observed in the entheses of our patients and the reduced pD signal often observed in larger joints, such as the knee.

In our study, achieving disease remission according to cDAPSA did not correlate significantly with the change in ultrasound scores, whereas achieving MDA correlated significantly only with the change in MIJET. Moreover, the change in ultrasound scores was not significantly associated with achieving values indicative of wellbeing in PROs measurements, except for the HAQ. This finding highlights the difference between clinical and ultrasound responses. Generally speaking, variations in MIJET and 2MIJET appear to correlate more with clinimetric indices than the GUIS score, although significance was reached in only a few items, likely due to the small sample size.

The statistical analysis of the distribution of mean ultrasound scores and clinimetric indices did not show significant differences between cResponders and non-cResponders in various assessments. This underscores that ultrasound and clinical variations are more reliable than individual baseline and subsequent assessments.

In our cohort, we noted a faster US response of MIJET and 2MIJET in cResponders treated with JAKi vs. TNFi and IL-17/12-23i, with a statistically significant difference already in Δ*t1-t0*. Conversely, differences in early clinical response according to cDAPSA in Δt1-t0 were not significant among these treatment groups, although they tended toward significance. Consequently, the difference in response speed between pharmacological classes was more pronounced from an ultrasound perspective than from a clinical standpoint (Figure 3).

In comparing the baseline characteristics of patients, non-cResponders had greater enthesitis involvement, with a higher mean of the LEI. This finding aligns with the literature suggesting that enthesitis-predominant sub-phenotype is associated with greater disease activity and is more refractory to treatment (18, 19). Furthermore, this data may be influenced by overlapping conditions such as fibromyalgia or chronic nociplastic pain, comorbidities that could complicate the identification of inflammatory pain, thereby impacting the perceived treatment response. The added value of US assessment and the MIJET and 2MIJET scores lies in their ability to detect subclinical inflammation that may not be identified through clinical examination, as well as to highlight the absence of ultrasound findings suggestive of enthesitis (20). In cases of predominant entheseal involvement, ultrasound evaluation of the enthesis at the site of pain localization—rather than the systematic assessment of predetermined entheses as conducted with the Leeds Enthesitis Index (LEI)—provides a more accurate understanding of the condition of the enthesis associated with pain, both at baseline and during treatment response assessment. This approach may help minimize the influence of potential confounding factors, thereby facilitating a more tailored therapeutic strategy targeting the underlying cause of pain. In our study, only 6 of the 29 patients had an associated diagnosis of fibromyalgia (4 cResponders and 2 non-cResponders), and the small sample size did not permit a subgroup analysis, which could have provided valuable insights.

No patients enrolled in the study had to discontinue treatment due to AE. It is noteworthy that the two cases of hypertensive crisis occurred in patients undergoing JAKi therapy. These two patients already had hypertension, and adjustments to their antihypertensive therapy were sufficient to achieve blood pressure control.

There are several limitations associated with this study. Firstly, the sample size was relatively small. Future studies with larger sample sizes would allow more accurate analysis, especially when comparing specific patient subgroups (e.g., different treatment approaches). Biologic-naïve patients were 5/22 among the cResponders and 1/7 among the non-cResponders; this difference, although not significant, may have influenced the assessment of clinical and ultrasound response. The heterogeneity of therapies prescribed to patients in our cohort may have influenced the evaluated outcomes. The US evaluation was performed by a single sonographer. There are also limitations in patient selection. Specifically, the presence of enthesitis or tenosynovitis at the time of selection was assessed clinically. Moreover, ours was a single-center study with a homogeneous Caucasian patient population.

## 5 Conclusion

In conclusion, our pilot study highlighted how US may be an early predictor of drug retention. We were able to identify statistically significant US changes 3 months after treatment initiation among cResponders vs. non-cResponders. The changes in US scores observed after 1 month were also greater among cResponders, though they did not reach significance.

Considering the impracticality of evaluating numerous joints included in the main composite US assessment scores, our results indicate that assessing only the most clinically involved joints can be equally valid in predicting treatment retention rates at 6 months. Furthermore, the variations in the US scores of MIJET and 2MIJET identified Responders more promptly compared to the unilateral GUIS.

Our pilot study highlighted how including US assessment in PsA trials could allow for a more timely and objective evaluation. Indeed, there is a distinction between US response and clinical response, with the latter being more influenced by comorbidities and secondary pain. Additionally, ultrasound response to therapy appears to differ across drug classes. Repeating similar studies but with a larger sample size could help develop more accurate precision medicine.

The identification of early predictors of treatment response may help mitigate the side effects and the overall financial burden on the healthcare system of ineffective therapies.

## Data Availability

The raw data supporting the conclusions of this article will be made available by the authors, without undue reservation.
