# Supplementary material for: Predictors of response to bDMARDs and tsDMARDs in psoriatic arthritis: a pilot study on the role of musculoskeletal ultrasound
Source: Front Med (Lausanne). 2024 Dec 23;11:1482894. doi: 10.3389/fmed.2024.1482894 (PMC11701151; doi:10.3389/fmed.2024.1482894)
Supplement: Supplementary file 1 [file Table_1.docx]

***Supplementary Table 1:*** Baseline characteristics of the included populations

| Characteristics | PsA patients n=29 |
| --- | --- |
| Age in years, mean (SD) | **57,72** (9,72) |
| Male sex, n (%) | **14** (48,3%) |
| Time since diagnosis of PsA in months, mean (SD) | **154** (101,85) |
| BMI, median (IQR) | **26,48** (23,48-29) |
| Smoking status, n (%)   - Smoker | **7** (24,1%) |
| - Ex-smoker | **5** (17,2%) |
| - Non-smoker | **17** (58,6%) |
| Onychopathy, n (%) | **12** (41%) |
| Prevalent Subtype of PsA, n (%)   - Polyarticular | **15** (51,7%) |
| - Oligoarticular | **7** (24,1%) |
| - Monoarticular | **3** (10,3%) |
| - Enthesitis | **4** (13,8%) |
| Disease activity | |
| Tender Joint Count (68 joints), mean (SD) | **10,28** (8,2) |
| Swollen Joint Count (66 joints), mean (SD) | **4,31** (4,27) |
| LEI, mean (SD) | **0,86** (1,76) |
| cDAPSA, mean (SD) | **28,16** (11,88) |
| HAQ, mean (SD) | **0,99** (0,58) |
| PsAID, mean (SD) | **4,55** (1,79) |
| Total PASI score, mean (SD) | **1,84** (3,21) |
| Treatment | |
| Biologic-naïve patients, n (%) | **6** (20,7%) |
| Multi-failure patient, n (%) | **15** (51,7%) |
| bDMARD/tsDMARD treatment, n (%)   - TNFi | **4** (13,8 %) |
| - IL17i | **10** (34,5 %) |
| - IL23/12-23i | **5** (17,2 %) |
| - Small molecules | **2** (6,9 %) |
| - No bDMARD/tsDMARD | **8** (27,6 %) |
| Concomitant csDMARD, n (%) | **7** (24,1 %) |
| Steroid (%) | **4** (13,8 %) |
| Blood tests |  |
| CRP (nr 0,5-5 mg/L), median (IQR) | **5,3** (1,89-10) |
| ESR (nr 2-30 mm/h), median (IQR) | **17,5** (6,25-27) |

**Legend:** SD= standard deviation; IQR= interquartile range; BMI= body mass index; PASI= Psoriasis Area Severity Index; LEI= leeds enthesitis index, HAQ= Health Assessment questionnaire; PsAID= Psoriatic Arthritis Impact of Disease; cDAPSA= clinical Disease Activity in PSoriatic Arthritis.
