# Supplementary material for: Predictors of response to bDMARDs and tsDMARDs in psoriatic arthritis: a pilot study on the role of musculoskeletal ultrasound
Source: Front Med (Lausanne). 2024 Dec 23;11:1482894. doi: 10.3389/fmed.2024.1482894 (PMC11701151; doi:10.3389/fmed.2024.1482894)
Supplement: Supplementary file 2 [file Table_2.docx]

***Supplementary Table* 2:** comparison of baseline demographic and clinical characteristics between cResponders and non-cResponders

| Characteristics | cResponder | non-cResponder | *p*-value |
| --- | --- | --- | --- |
| Age in years, mean | 57,68 | 51,33 | 0,2536 |
| Male, n (%) | 10 (45%) | 4 (57%) | 0.681 |
| Time since diagnosis of PsA in months,mean | 160,3 | 136,3 | 0,5852 |
| BMI (kg/m2) | 27 | 26,1 | 0,2854 |
| Smoking (ex-smoker and smoker/no smoker) | 7 (31,8%) | 5 (71,4%) | 0.0921 |
| Onychopathy, yes/no | 7 (31,8%) | 5 (71,4%) | 0,0924 |
| Tender Joint Count (68 joint), mean | 9,364 | 13,14 | 0,2882 |
| Swollen Joint Count (66 joints), mean | 4,227 | 4,571 | 0,8551 |
| PASI, mean | 1,432 | 3,14 | 0,2178 |
| LEI, mean | **0,41** | **2,29** | **0,0095*** |
| cDAPSA, mean | 26,95 | 32 | 0,3274 |
| HAQ, mean | 0,96 | 1,07 | 0,6826 |
| PsAID, mean | 4,22 | 5,55 | 0,0834 |
| Biologic-naïve patients, yes/no | 5 (22,7%) | 1 (14,29%) | 0,6457 |

**Legend:** BMI= body mass index; PASI= Psoriasis Area Severity Index; LEI= leeds enthesitis index, HAQ= Health Assessment questionnaire; PsAID= Psoriatic Arthritis Impact of Disease; cDAPSA= clinical Disease Activity in PSoriatic Arthritis; *= statistically significant variations.
