# Supplementary material for: Predictors of response to bDMARDs and tsDMARDs in psoriatic arthritis: a pilot study on the role of musculoskeletal ultrasound
Source: Front Med (Lausanne). 2024 Dec 23;11:1482894. doi: 10.3389/fmed.2024.1482894 (PMC11701151; doi:10.3389/fmed.2024.1482894)
Supplement: Supplementary file 3 [file Table_3.docx]

***Supplementary Table* 3:** t-test results for the correlation between clinimetric indices and the variation in ultrasound scores at t6.

| *Δt6-t0 MIJET* | | | | | | | | | | | | | | | | | | | | |
| --- | --- | --- | --- | --- | --- | --- | --- | --- | --- | --- | --- | --- | --- | --- | --- | --- | --- | --- | --- | --- |
| gVAS≤1 | **gVAS**  **>1** | **p-value** | **pVAS**  **≤1** | **pVAS >1** | **p-value** | **MS ≤1** | **MS >1** | **p-value** | **cDAPSA ≤4** | **cDAPSA >4** | **p-value** | **MDA**  **≤1** | **MDA >1** | **p-value** | **HAQ ≤1** | **HAQ >1** | **p-value** | **PsAID≤1** | **PsAID>1** | **p-value** |
| -2,167 | -1,087 | 0,1895 | -2,167 | -1,167 | 0,2220 | -1,455 | -1,158 | 0,6643 | -2 | -1,167 | 0,3488 | **-2,5** | **-0,8571** | **0,0229** | **-4,667** | **-0,9231** | **0,0001** | -2,6 | -1,042 | 0,0734 |
| *Δt6-t0 2MIJET* | | | | | | | | | | | | | | | | | | | | |
| gVAS≤1 | **gVAS**  **>1** | **p-value** | **pVAS**  **≤1** | **pVAS >1** | **p-value** | **MS ≤1** | **MS >1** | **p-value** | **cDAPSA ≤4** | **cDAPSA >4** | **p-value** | **MDA**  **≤1** | **MDA >1** | **p-value** | **HAQ ≤1** | **HAQ >1** | **p-value** | **PsAID≤1** | **PsAID>1** | **p-value** |
| -3,333 | -2,348 | 0,4702 | -4 | -2,125 | 0,1563 | -2,4 | -2,714 | 0,7877 | -3,4 | -2,375 | 0,4838 | -4,125 | -1,952 | 0,0715 | **-6,33** | **-2,115** | **0,0145** | -3,6 | -2,333 | 0,3857 |
| *Δt6-t0 GUIS* | | | | | | | | | | | | | | | | | | | | |
| gVAS≤1 | **gVAS**  **>1** | **p-value** | **pVAS**  **≤1** | **pVAS >1** | **p-value** | **MS ≤1** | **MS >1** | **p-value** | **cDAPSA ≤4** | **cDAPSA >4** | **p-value** | **MDA**  **≤1** | **MDA >1** | **p-value** | **HAQ ≤1** | **HAQ >1** | **p-value** | **PsAID≤1** | **PsAID>1** | **p-value** |
| *-3* | *-3,625* | *0,8325* | *-4,167* | *-3,333* | *0,7778* | *-5,111* | *-3,158* | *0,4638* | *-2,400* | *-3,875* | *0,6468* | *-4,250* | *-3,381* | *0,7496* | *-8,667* | *-3,038* | *0,1515* | *-3,800* | *-3,583* | *0,9464* |

**Legend:** MIJET=Most Involved Joint/Enthesis/Tendon; 2MIJET= two Most Involved Joints/Entheses/Tendons; GUIS= Global US Inflammation Subscore; gVAS= global assessment visual analog scale; pVAS= pain visual analog scale; MS= morning stiffness; cDAPSA= clinical disease activity index for psoriatic arthritis; MDA=minimal disease activity; HAQ=health assessment questionnaire; PsAID= psoriatic arthritis impact of disease.
