# Supplementary material for: Predictors of response to bDMARDs and tsDMARDs in psoriatic arthritis: a pilot study on the role of musculoskeletal ultrasound
Source: Front Med (Lausanne). 2024 Dec 23;11:1482894. doi: 10.3389/fmed.2024.1482894 (PMC11701151; doi:10.3389/fmed.2024.1482894)
Supplement: Supplementary file 4 [file Table_4.docx]

***Supplementary Table* 4:** density plots of the trends in the mean values of clinimetric and ultrasound indices between cResponders and non-cResponders at t0, t1, t3

|  | p-value |
| --- | --- |
| GUIS | 0,325879472 |
| GUIS.e | 0,497143864 |
| MIJET | 0,348527457 |
| 2MIJET | 0,284539495 |
| pVAS | 0,409746405 |
| MS | 0,335956052 |
| cDAPSA | 0,284077769 |
| HAQ | 0,474997303 |
| PsAID | 0,429231062 |

**Legend:** MIJET=Most Involved Joint/Enthesis/Tendon; 2MIJET= two Most Involved Joints/Entheses/Tendons; GUIS= Global US Inflammation Subscore; GUIS.e= Global US Inflammation Subscore enthesis; pVAS= pain visual analog scale; MS= morning stiffness; cDAPSA= clinical disease activity index for psoriatic arthritis; HAQ=health assessment questionnaire; PsAID== psoriatic arthritis impact of disease.
