# Supplementary material for: Predictors of response to bDMARDs and tsDMARDs in psoriatic arthritis: a pilot study on the role of musculoskeletal ultrasound
Source: Front Med (Lausanne). 2024 Dec 23;11:1482894. doi: 10.3389/fmed.2024.1482894 (PMC11701151; doi:10.3389/fmed.2024.1482894)
Supplement: Supplementary file 5 [file Table_5.docx]

***Supplementary Table 5*:** The mean variations of values between *t1*-*t0*, *t3*-*t0*, and *t6*-*t0* among c-Responder patients across different pharmacological classes

|  | *Δt1-t0* | | | *Δt3-t0* | | | *Δt6-t0* | | |
| --- | --- | --- | --- | --- | --- | --- | --- | --- | --- |
|  | TNFi | IL-17/12/23i | p-value | TNFi | IL-17/12/23i | p-value | TNFi | IL-17/12/23i | p-value |
| GUIS | -0,8472 | -1,300 | 0,7625 | -2,667 | -3,100 | 0,8654 | -3,222 | -3,100 | 0,9605 |
| MIJET | -0,4444 | -0,1000 | 0,5743 | -0,7778 | -1,500 | 0,2378 | -0,6667 | -1,700 | 0,0983 |
| 2MIJET | -1,111 | -0,3000 | 0,2353 | -2,222 | -2,300 | 0,9533 | -2,222 | -2,600 | 0,7748 |
| cDAPSA | -7,889 | -6,900 | 0,8071 | -10,11 | -9,500 | 0,8960 | -11,78 | -7,300 | 0,3001 |
|  | TNFi | JAKi | p-value | TNFi | JAKi | p-value | TNFi | JAKi | p-value |
| GUIS | -0,8472 | -3,600 | 0,2119 | -2,667 | -3,400 | 0,7626 | -3,222 | -8,200 | 0,1838 |
| MIJET | -0,4444 | -2,600 | **0,0491*** | -0,7778 | -2,800 | 0,1142 | -0,6667 | -3,200 | **0,0126*** |
| 2MIJET | -1,111 | -4,200 | **0,0333*** | -2,222 | -4,800 | 0,1734 | -2,222 | -5,200 | 0,0699 |
| cDAPSA | -7,333 | -15,20 | 0,0586 | -10,11 | -14,60 | 0,2398 | -11,78 | -17,80 | 0,1983 |
|  | IL-17/12/23i | JAKi | p-value | IL-17/12/23i | JAKi | p-value | IL-17/12/23i | JAKi | p-value |
| GUIS | -1,300 | -3,600 | 0,1992 | -3,100 | -3,400 | 0,9348 | -3,100 | -8,200 | 0,2691 |
| MIJET | -0,1000 | -2,600 | **0,0194*** | -1,500 | -2,800 | 0,2535 | -1,700 | -3,200 | 0,1644 |
| 2MIJET | -0,3000 | -4,200 | **0,0021*** | -2,300 | -4,800 | 0,1583 | -2,600 | -5,200 | 0,1094 |
| cDAPSA | -6,900 | -15,20 | 0,0635 | -9,500 | -14,60 | 0,3614 | -7,300 | -17,80 | 0,0699 |

**Legend:** GUIS= Global US Inflammation Subscore; MIJET=Most Involved Joint/Enthesis/Tendon; 2MIJET= two Most Involved Joints/Entheses/Tendons; cDAPSA= clinical Disease Activity in PSoriatic Arthritis; *= statistically significant variations.
